# Feasibility of supported self-management with a pictorial action plan to improve asthma control

**DOI:** 10.1038/s41533-022-00294-8

**Published:** 2022-09-20

**Authors:** Shariff Ghazali Sazlina, Ping Yein Lee, Ai Theng Cheong, Norita Hussein, Hilary Pinnock, Hani Salim, Su May Liew, Nik Sherina Hanafi, Ahmad Ihsan Abu Bakar, Chiu-Wan Ng, Rizawati Ramli, Azainorsuzila Mohd Ahad, Bee Kiau Ho, Salbiah Mohamed Isa, Richard A. Parker, Andrew Stoddart, Yong Kek Pang, Karuthan Chinna, Aziz Sheikh, Ee Ming Khoo, RESPIRE collaboration, Shariff Ghazali Sazlina, Ee Ming Khoo, Hilary Pinnock, Aziz Sheikh

**Affiliations:** 1 Department of Family Medicine, Faculty of Medicine and Health Sciences, Universiti Putra Malaysia, Serdang, Selangor Malaysia; 2 Malaysian Research Institute on Ageing (MyAgeing™), Universiti Putra Malaysia, Serdang, Malaysia; 3 UMeHealth Unit, Faculty of Medicine, University of Malaya, Kuala Lumpur, Malaysia; 4 Department of Primary Care Medicine, Faculty of Medicine, University of Malaya, Kuala Lumpur, Malaysia; 5 NIHR Global Health Research Unit on Respiratory Health (RESPIRE), Usher Institute, The University of Edinburgh, Edinburgh, UK; 6 Hospital Pusrawi Sdn Bhd, Kuala Lumpur, Malaysia; 7 Centre for Epidemiology and Evidence-Based Practice, Department of Social and Preventive Medicine, Faculty of Medicine, University of Malaya, Kuala Lumpur, Malaysia; 8 Klinik Kesihatan Lukut, Ministry of Health Malaysia, Port Dickson, Negeri Sembilan Malaysia; 9 Klinik Kesihatan Bandar Botanik, Ministry of Health Malaysia, Klang, Selangor Malaysia; 10 Edinburgh Clinical Trials Unit, Usher Institute, The University of Edinburgh, Edinburgh, UK; 11 Department of Medicine, Faculty of Medicine, University of Malaya, Kuala Lumpur, Malaysia; 12 Faculty of Business and Management, UCSI University, Kuala Lumpur, Malaysia; 13 Department of Family Medicine, Faculty of Medicine and Health Sciences, Universiti Putra Malaysia, Serdang, Selangor Malaysia; 14 Malaysian Research Institute on Ageing (MyAgeing™), Universiti Putra Malaysia, Serdang, Malaysia; 15 Department of Primary Care Medicine, Faculty of Medicine, University of Malaya, Kuala Lumpur, Malaysia; 16 NIHR Global Health Research Unit on Respiratory Health (RESPIRE), Usher Institute, The University of Edinburgh, Edinburgh, UK

**Keywords:** Patient education, Outcomes research

## Abstract

Supported self-management reduces asthma-related morbidity and mortality. This paper is on a feasibility study, and observing the change in clinical and cost outcomes of pictorial action plan use is part of assessing feasibility as it will help us decide on outcome measures for a fully powered RCT. We conducted a pre–post feasibility study among adults with physician-diagnosed asthma on inhaled corticosteroids at a public primary-care clinic in Malaysia. We adapted an existing pictorial asthma action plan. The primary outcome was asthma control, assessed at 1, 3 and 6 months. Secondary outcomes included reliever use, controller medication adherence, asthma exacerbations, emergency visits, hospitalisations, days lost from work/daily activities and action plan use. We estimated potential cost savings on asthma-related care following plan use. About 84% (*n* = 59/70) completed the 6-months follow-up. The proportion achieving good asthma control increased from 18 (30.4%) at baseline to 38 (64.4%) at 6-month follow-up. The proportion of at least one acute exacerbation (3 months: % difference −19.7; 95% CI −34.7 to −3.1; 6 months: % difference −20.3; 95% CI −5.8 to −3.2), one or more emergency visit (1 month: % difference −28.6; 95% CI −41.2 to −15.5; 3 months: % difference −18.0; 95% CI −32.2 to −3.0; 6 months: % difference −20.3; 95% CI −34.9 to −4.6), and one or more asthma admission (1 month: % difference −14.3; 95% CI −25.2 to −5.3; 6 months: % difference −11.9; 95% CI −23.2 to −1.8) improved over time. Estimated savings for the 59 patients at 6-months follow-up and for each patient over the 6 months were RM 15,866.22 (USD3755.36) and RM268.92 (USD63.65), respectively. Supported self-management with a pictorial asthma action plan was associated with an improvement in asthma control and potential cost savings in Malaysian primary-care patients.

*Trial registration number:* ISRCTN87128530; prospectively registered: September 5, 2019, http://www.isrctn.com/ISRCTN87128530.

## Introduction

Asthma affects almost 300 million people globally and 100 million people in Southeast Asia^1,2^. Most asthma-related deaths occur in low- and middle-income countries^2^. Annually in Malaysia, 68% of people with asthma visit their doctor; 50% attend emergency department, and 10% are admitted, this representing substantial morbidity and incurring substantial emergency healthcare costs^3,4^. Only a third of people with asthma attend regular follow-up care with low usage of controller medications and overuse of oral short-acting beta-agonist^5–7^. Despite this, asthma is not a healthcare priority in Malaysia compared to other non-communicable diseases (cardiovascular diseases and diabetes) and is relatively underfunded^8^.

All major national and international asthma guidelines recommend asthma self-management that is personalised to patients’ preferences and views^9,10^ to improve clinical outcomes and reduce healthcare costs^11,12^. Asthma action plans are an integral component of supported self-management in which patients are given written advice on how to adjust their treatment according to changes in their disease status^9,10,13^. However, in Malaysia, about 60% of adults with asthma have limited health literacy challenging use of traditional text-based plan^14^. An action plan that provides guidance in a pictorial format has the potential to overcome inequities^15–18^ for people with limited literacy and numeracy skills by making complex health information easier to comprehend^19^, and beneficial^20^.

Several studies have reported pictorial asthma action plan for use in adults^20–22^. Roberts et al. have developed a validated pictorial asthma action plan that was comprehensible in three different populations of asthma patients, including Malaysia^20^. Two controlled trials using validated pictorial action plans have yielded contrasting findings^21,22^. A non-randomised controlled trial among ‘illiterate’ women with asthma in Turkey showed that pictorial action plans improved asthma control and quality of life^21^ whilst a randomised controlled trial (*n* = 62) in a semi-urban primary-care clinic in Malaysia found no significant difference in asthma control between patients who received a pictorial or text-based action plan^22^. In the Malaysian study, the participants had relatively well-controlled asthma at baseline, potentially reducing the scope for improvement. In addition, the pictorial plan used in the study did not align with the advice of the Malaysian asthma guideline.

A previous study on delivering supported self-management in the context of Malaysian primary care highlighted that the written action plan endorsed by the Malaysian Thoracic Society^10^ was not understood by patients possibly because of limited health literacy combined with the language challenges of living in a multilingual society^23^. We have explored this issue in some detail^24^ and found that a written action plan is a particularly an important barrier in Malaysia, hence, the need to explore the role of a pictorial action plan in asthma-supported self-management.

We therefore aimed to determine the feasibility of providing a pictorial action plan for adult patients with asthma and estimate its potential impact on asthma control, medication use, healthcare utilisation, costs and days lost from work or usual activities as well as the feasibility of assessing costs related to asthma care. Our findings will inform the design of a future randomised controlled trial.

## Methods

### Study design and setting

Embedded within the Medical Research Council framework for design and evaluation of complex interventions^25^, this pre–post feasibility study was conducted in an urban public primary-care clinic in the district of Klang, Selangor, Malaysia between September 2019 to July 2020. The study protocol was registered with BMC ISRCTN Registry [ISRCTN87128530; prospectively registered: September 5, 2019, http://www.isrctn.com/ISRCTN87128530]. The state of Selangor was chosen as it has a high prevalence of adults with asthma (22%), especially in urban communities as well as the highest prevalence of limited health literacy in Malaysia at 75%^26^.

### Participants

The study participants were recruited from one of the primary-care clinics under the Klang Asthma Cohort (KAC) registry (a clinical asthma patients registry) using an Excel-generated simple random table by a research member, based on the inclusion and exclusion criteria in Table 1. Klang Asthma Cohort is a cohort of 1280 people with asthma recruited from six primary-care clinics in Klang who are willing to be approached for future research. A detailed description of KAC can be accessed at https://www.ed.ac.uk/usher/respire/chronic-respiratory-disorders/asthma-care.

**Table 1:** Participant eligibility criteria.

No.	Eligibility criteria
1.	**Inclusion criteria** 1 Aged 18 years or older under follow-up care of the asthma clinic. 2 Asthma diagnosed by a healthcare practitioner. 3 Prescribed daily inhaled corticosteroids (ICS) for poor asthma control in the last year (according to Global Initiative for Asthma (GINA) Asthma Symptoms Control (2019) step 2 management for asthma control), in addition to as-needed inhaled short-acting beta_2_-agonist (SABA); or as-needed low dose ICS-long acting beta_2_-agonist (LABA) for those on SMART therapy^9^. 4 Able to provide informed consent. 5 Able to understand Malay (national language of Malaysia) or English.
2.	**Exclusion criteria** 1 Co-morbid conditions prohibiting participation, such as cognitive impairment. 2 Other diagnosed chronic respiratory disease (e.g. chronic obstructive pulmonary disease).

Participants were contacted via a telephone call (to avoid written communication in people with limited literacy) by a trained research assistant who provided a detailed description of the study. Those who agreed to participate in the study met face-to-face with the research assistant at the clinic to provide written informed consent and to answer the baseline questionnaire.

As this was a feasibility study, a formal sample size calculation was not required. Seventy participants were recruited, which was deemed to be adequate to inform the feasibility of delivering the intervention^27^.

### Usual clinic care and self-management support

The selected primary-care clinic has a dedicated asthma clinic that operates one afternoon a week involving medical officers, pharmacists and nurses. Medical officers are doctors without postgraduate training who work in primary-care clinics under the leadership of specialist family physicians. They are trained to assess asthma control, check the use of peak expiratory flow rate, recommend appropriate treatment, and deliver supported self-management including a text-based asthma action plan, and as the participants’ usual doctor, continued to provide care throughout this study. The pharmacists taught inhaler technique and discussed adherence to medications and asthma action plans. The nurses provided asthma education. For participants in this feasibility study, the clinic management continued as usual, but a pictorial asthma action plan was provided instead of the standard text-based action plan.

### Intervention

The intervention consisted of a pictorial asthma action plan (see Supplementary Fig. 1) instead of the text-based action plan incorporated within the existing self-management education and support, and is described in Table 2 using the Template for Intervention Description and Replication (TIDieR) checklist^28^.

**Table 2:** TIDieR Checklist.

TIDieR item	Description
Title	Supported self-management using pictorial asthma action plan.
Why	This study addressed components of the COM-B Behaviour Change Wheel ^45^ to improve asthma control by supporting self-management behaviour, including the use of a pictorial asthma action plan personalised to the patient’s capability, motivation and opportunity. • Capability: Psychological and physical capacity to use the plan were considered and the self-management support personalised accordingly. • Opportunity: Use of a pictorial action plan providing the opportunity for participants with limited health literacy to understand and use the action plan. • Motivation: Supported self-management strategies were provided to enhance motivation.
What	We adapted the format of pictorial asthma action plan from the plan used in published studies^20,22^ and aligned the advice with the (text) action plan of the Malaysian Management of Asthma in Adults guidelines^10^. Adaptation was an iterative process that involving an advisory group comprising two doctors from the clinic, and four patient and public involvement (PPI) colleagues with asthma (who had differing experience of using text-based asthma action plans) and two relatives of patients with asthma. • The pictorial asthma action plan illustrated different levels of asthma control with pictures to depict asthma symptoms and zoned actions needed such as adjusting the dose of reliever, use of prednisolone or seeking medical attention (Fig. 1). The characters in the pictures were used following the feedback from the patient and public to represent the range of ethnic groups in Malaysia. The action plan was developed in English language and translated to Malay language, using the forward and backward translation processes. • The pictorial action plan in both languages had undergone content validity checks by nine panellists comprising five healthcare providers involved in the management of asthma in primary-care facilities and four patients with asthma who had used a written asthma action plan. They commented on (1) accuracy (the pictures conveyed the intended meaning); (2) clarity (the pictures were understood and provided clear information about zone of asthma care); (3) style (font and picture size were appropriate); and (4) relevance (the pictures were relevant to the local social context). • Using the Content Validity Ratio^46^, the action plan was considered valid for use in the Malaysian context (see Supplementary Information for details of content validation process) with the exception of one picture depicting wheeze which was considered unclear by one panellist. Based on this feedback, we added the word ‘wheeze’ below the picture.
Who provided	The action plan was provided by the clinic’s medical officers and adherence to the plan discussed with the pharmacists. • The research team conducted a 2-h group training for the clinic’s healthcare providers (medical officers, pharmacists and nurses) during a scheduled Continuous Professional Development (CPD) session which aims to maintain staff skills. The training emphasised communication skills and included interactive lectures, role-plays using simulated patient consultations and group discussion to familiarise the staff with the pictorial action plan as compared to the written plan with which they were familiar.
How	The action plan was personalised for each participant and was provided one-to-one by the clinic’s medical officers and assisted by the pharmacists.
Where	The action plan was provided at the dedicated asthma clinic run routinely at the public primary-care clinic.
When and How much	The intervention (provision of the pictorial action plan) was provided after the baseline assessment during the participants’ scheduled clinic visits. • They were taught how to use the action plan by the medical officers at the first visit. • Pharmacists then discussed adherence to action plan use.
Tailoring	The action plan was personalised for each participant such as type of controller medication and medication dosage. The doctors who provided the action plan would circle a picture of the relevant controller medication and write down the dosage to be taken by the participant.
Fidelity assessment	During the first clinic visit, the research team checked whether all participants had received a pictorial action plan that was completed with relevant information. Relevance was judged independently by two primary-care doctors who discussed disagreements to reach consensus.

### Outcome measures

All study outcomes were measured at baseline and at 1-, 3- and 6 months post intervention as in the questionnaire (see Supplementary Information: Questionnaire). We initially intended to follow up the participants over 12 months but had to stop data collection at 6 months to comply with restrictions during the COVID-19 pandemic.

Asthma control was the primary outcome and measured using the validated Global Initiative for Asthma (GINA) Asthma Symptoms Control^9^. This questionnaire comprises four questions that measure the adequacy of asthma treatment in the past four weeks. The questions focus on the day and night-time symptoms, use of reliever, and limitation of activity due to asthma. The option for each response is either 'Yes' or 'No'. Well-controlled was considered if the responses to all questions were 'No'. Any responses of 'Yes' were considered as not controlled.

The secondary outcomes measured in this study all related to the previous 1 month:

- Number of times reliever medication (inhaled or oral bronchodilators) was used
- Adherence to controller medication
- Frequency of acute exacerbations (defined as episodes characterised by acute or subacute onset of progressively worsening symptoms, such as shortness of breath, cough, wheezing or chest tightness, which are worse than the patient's usual status and require a change in treatment)
- Frequency of asthma-related emergency visits (to a health clinic and/or hospital emergency department)
- Frequency of asthma-related admissions
- Numbers of days lost from work for asthma treatment (defined as the number of days of medical leave taken by an employee, or unable to work if self-employed)
- Number of times the participants reported using their pictorial asthma action plan in the previous month.

### Data collection

Data were collected face-to-face using a pretested structured questionnaire in English or Malay language. At baseline, there were four sections to the questionnaire:

Section 1: Socio-demographic and socio-economic information, including age, gender, ethnicity, highest education level, occupation, marital status, personal and household incomes.

Section 2: Medical and healthcare information, including duration of asthma, triggers and allergies, frequency of attacks, use of healthcare resources, medications, vaccinations, current and history of alternative treatment use, smoking status, co-morbid conditions, previous asthma education and ownership/use of an asthma action plan, use of an asthma diary.

Section 3: Asthma control assessment using the GINA Asthma Symptom Control.

Section 4: Health literacy was measured using the validated 47-item Asian version of the Health Literacy Survey-Asia-Q47 (HLS-ASIA-Q47) which assesses the ability to access, understand, appraise, and use health information in the context of healthcare, disease prevention, and health promotion^29^. The HLS-Asia-Q47 has been shown to be valid and reliable for use in Malaysia^30^. It was rated on 4-Likert scale, ranged from 1 = very difficult to 4 = very easy. According to the instructions with the HLS-ASIA-Q47, an index of health literacy score was constructed using the mean-based scores of the 47 items. These were transformed into a unified metric ranging from 0 to 50 using the formula = (*mean* – 1)* (50/3)^31^. The index scores were grouped into two categories: limited and adequate health literacy. An index score of ≤33 indicates limited health literacy^31^.

Information on healthcare visits (emergency visits at the clinic for attacks) were verified by clinic doctors from the participants’ medical records. In case of any discrepancies, the information was checked with the patients, as patients in Malaysia might seek care from other health providers, and the medical record may not be complete.

Follow-up data on all the primary and secondary outcomes were collected at 1-, 3- and 6-month post intervention by trained enumerators who were medical doctors not involved in patients’ recruitment and baseline assessments. At every follow-up visit, primary and secondary outcomes were collected, and participants asked about reasons for using a pictorial action plan and any barriers and facilitators.

### Data analysis

We used IBM SPSS version 26.0^32^ and R software version 4.0.4^33–35^, for the statistical analysis. Descriptive analysis of the baseline variables was reported using means and standard deviations for continuous variables and frequencies and percentages for categorical data. Chi-squared or Fisher’s Exact tests (for small numbers) for categorical variables and independent samples *t* test for continuous data were used to compare the difference in baseline characteristics between the participants who had completed, withdrawn or lost to follow-up.

The primary and secondary outcomes were categorised as binary data. We calculated the difference in paired percentages with well-controlled asthma, no reliever use, at least one missed day using controller medication, at least one acute exacerbation, at least one emergency visit, and at least one admission, for each of the follow-up time points compared to baseline. The analysis was completed using all data available with no imputation made for missing data (i.e., missing data were left as missing). The 'modified Wilson score method' or 'Newcombe score method' was used to calculate the Exact 95% confidence intervals for all paired differences.

### Feasibility of assessing the cost of asthma-related care

An expert panel comprising a Ministry of Health (MoH) family medicine specialist and a pharmacist, and the research team who were family medicine specialists and a respiratory physician, reached consensus on the annual cost of care for a person with well-controlled and uncontrolled asthma. The unit costs of specialist and general outpatient visits were obtained from the legislated fee schedules for the Ministry of Health, Malaysia services which reflect the actual cost of services^36^. The fee schedule details fees for MoH facilities for non-citizens who were not eligible for subsidised healthcare in Malaysia. Thus, the fees are the estimated cost of care in public health facilities in the country. We estimated the cost savings over six months for the participants who completed the study. This estimation was based on the differences between the estimated cost incurred in the absence of the intervention and the actual costs as observed. However, resource use had only been captured for 3 months out of the 6-months follow-up (for months 1, 3 and 6 during the follow-up at 1 month, 3 months and 6 months). Therefore, in order to estimate medication costs, it was assumed that (a) asthma status at baseline remained throughout month 1; (b) asthma status at 1-month follow-up remained for months 2 and 3; and (c) asthma status at the 3-month follow-up remained for months 4, 5 and 6. The details are discussed in the Supplementary Information: Cost of asthma-related care and Supplementary Table 1.

### Ethics approval

Regulatory approvals have been obtained in line with the operating procedures of the RESPIRE Global Unit, including approvals from the National Medical Research Ethics Committee, Ministry of Health, Malaysia [NMRR-18-2683-43494] and relevant authorities involved in the Klang District. Both verbal and written informed consent were obtained from eligible participants before the involvement of this study. Confidentiality of the participants was ensured; data were anonymised before publication or report writing. The study was conducted in accordance with the principles of the International Conference on Harmonisation Tripartite Guideline for Good Clinical Practice. This study also received sponsorship approval from the Academic and Clinical Central Office for Research & Development (ACCORD) at the University of Edinburgh.

### Reporting summary

Further information on research design is available in the Nature Research Reporting Summary linked to this article.

## Results

**Fig. 1:**
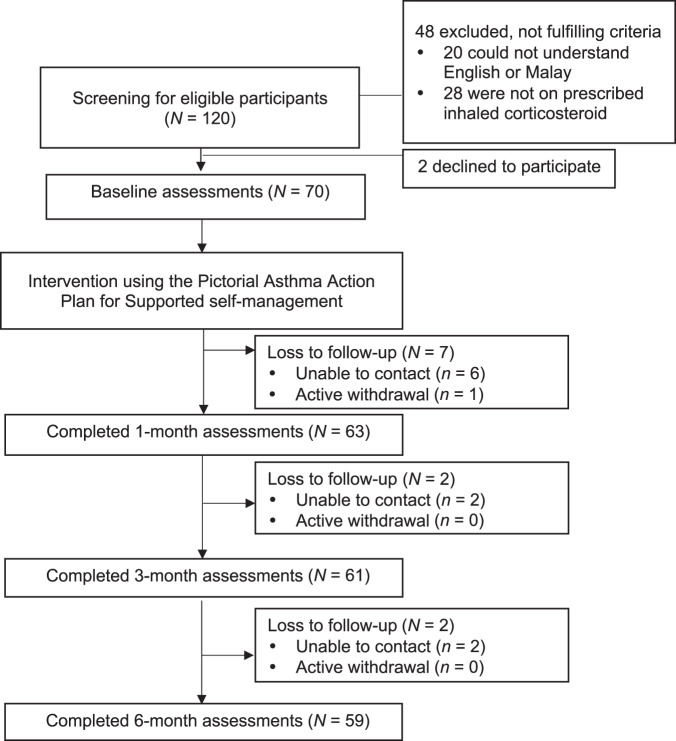
Flow of study participants.

Figure 1 summarises the flow of the participants in the study. We identified and screened 120 adult patients with asthma from the KAC registry for eligibility between June and August 2019. Of these, 72 eligible participants were recruited, two withdrew immediately after recruitment for personal reasons, therefore, 70 participants were enrolled in this study and attended the baseline clinic visits between September and November 2019. Of the 70 participants, 59 (84.3%) completed a 6-month follow-up. Table 3 compares the participants who completed the study and those who lost to follow-up. Those who lost to follow-up had higher personal income than those who completed the study (*P* = 0.019).

**Table 3:** Comparisons between participants who completed the study and those lost to follow-up.

Variables	Total, *N* = 70	Completed study, *N* = 59	Lost to follow-up, *N* = 11	*P* values
Age	51.2±15.5*	52.03±15.98*	46.55±11.82*	0.283
Gender^a^ (*n* (%))				
Men	29 (41.4)	24 (40.7)	5 (45.5)	0.768
Women	41 (58.6)	35 (59.3)	6 (54.5)	
Ethnicity^b^ (*n* (%))				
Malay	30 (42.9)	24 (40.7)	6 (54.5)	0.663
Indian	34 (48.6)	30 (50.8)	4 (36.4)	
Chinese and others	6 (8.6)	5 (8.5)	1 (9.1)	
Highest education^a^ (*n* (%))				0.775
No formal and primary level	15 (21.4)	13 (22.0)	2 (18.9)	
Secondary level and higher	55 (78.6)	46 (78.0)	9 (81.1)	
Occupation^a^ (*n* (%))				0.050
Retired/not working	38 (54.3)	35 (59.3)	3 (27.3)	
Working	32 (45.7)	24 (40.7)	8 (72.3)	
Marital status^a^ (*n* (%))				0.454
Married	51 (72.9)	44 (74.6)	7 (63.6)	
Unmarried	19 (27.1)	15 (25.4)	4 (36.4)	
Personal income (RM) (mean (SD))	1398.02 (1342.40)*	1230.00 (1240.00)*	2300.00 (1574.00)*	0.019**
	(USD317.78 (305.15))	(USD279.60 (281.87))*	(USD522.84 (357.80))*	
Household income (RM) (mean (SD))	3072.11 (2769.77)*	3002.00 (3409.00)*	3409.00 (3033.00)*	0.661
	(USD698.35 (629.62))	(USD682.43 (774.95))*	(USD774.95 (689.43))*	
Health literacy (Asian-HLS)^a^ (*n* (%))				0.609
Adequate	27 (38.6)	22 (37.3)	5 (45.5)	
Limited	43 (61.4)	37 (62.7)	6 (54.5)	

^a^Fisher’s Exact test; ^b^Chi-square test; *mean ± standard deviation; USD1= RM4.40 (on June 20, 2022); **P < 0.05 = statistically significant.

### Baseline characteristics

The mean age of the participants was 51.2 ± SD15.5 years, 58.6% were women, 72.3% were married and 48.6% were Indian, 42.9% were Malay and 8.6% with Chinese and other ethnicities. The majority (91.4%) of the participants had previously received supported self-management asthma education from the clinic staff, 25 (35.7%) had a written action plan of whom only 60% had used the plan. Even though 54 (77.1%) had an asthma diary, only 14 of them used it. About one-fifth of the participants used complementary and alternative medicines. In terms of health literacy, 61.4% had limited literacy. Only 24.3% used a smartphone for asthma information.

### Asthma control

There was an increasing proportion of well-controlled asthma from baseline to 6-month follow-up. In addition, there was an improvement in the proportion of controlled asthma at three months (% difference = 26.2; 95% confidence interval (CI) = 9.3, 41.1) and at 6 months (% difference = 33.9; 95% CI = 18.6, 46.7) post intervention compared to baseline as shown in Table 4.

**Table 4:** Percentage differences on well-controlled asthma over time.

Comparison with baseline	Baseline, *N* (%)	Follow-up, *N* (%)	Percentage difference	Lower 95% CI limit	Upper 95% CI limit
*Well-controlled asthma compared to baseline*					
Baseline	24/70 (34.3)				
1 month	19/63 (30.2)	27 (42.8)	12.7%	−0.8%	25.5%
3 months	18/61 (29.5)	34 (55.7)	26.2%	9.3%	41.1%
6 months	18/59 (30.5)	38 (64.4)	33.9%	18.6%	46.7%

*CI* confidence interval.

### Secondary outcomes

As shown in Table 5, the proportion of those with at least one acute exacerbation in the previous month reduced by 20% at the 3-month and 6-month follow-up compared to baseline. Further, the proportion of at least one emergency visit in the previous month reduced by 18% and 20% at the 3-month and 6-month follow-ups. Similarly, the proportion who had been hospitalised in the previous month reduced by 12% in the month prior to the 6-month follow-up compared to baseline.

**Table 5:** Percentage differences on secondary study outcomes over time.

Comparison with baseline	Baseline, *N* (%)	Follow-up, *N* (%)	Percentage difference	Lower 95% CI limit	Upper 95% CI limit
No reliever medication use compared to baseline					
Baseline	19/70 (27.1)				
1 month	15/63 (23.8)	18 (28.8)	4.8%	−8.6%	28.6%
3 months	15/61 (24.6)	22 (36.1)	11.5%	−5.1%	27.2%
6 months	14/59 (23.7)	21 (35.6)	11.9%	−3.1%	26.1%
At least one missed day using controller medication compared to baseline					
Baseline	34/70 (48.6)				
1 month	32/63 (50.7)	28 (44.4)	−6.3%	−20.8%	8.6%
3 months	29/61 (47.5)	25 (40.9)	−6.8%	−20.7%	7.7%
6 months	30/59 (50.8)	31 (52.5)	1.7%	−14.0%	17.3%
At least one acute exacerbation compared to baseline					
Baseline	39/70 (55.7)				
1 month	32/63 (50.8)	28 (44.4)	−6.3%	−22.0%	9.8%
3 months	31/61 (50.8)	19 (31.1)	−19.7%	−34.7%	−3.1%
6 months	30/59 (50.8)	18 (30.5)	−20.3%	−35.8%	−3.2%
At least one emergency visit compared to baseline					
Baseline	26/70 (37.1)				
1 month	22/63 (34.9)	4 (6.3)	−28.6%	−41.2%	−15.5%
3 months	20/61 (32.8)	9 (14.8)	−18.0%	−32.2%	−3.0%
6 months	20/59 (33.9)	8 (13.6)	−20.3%	−34.9%	−4.6%
At least one hospital admission compared to baseline					
Baseline	11/70 (15.7)				
1 month	9/63 (14.3)	0	−14.3%	−25.2%	−5.3%
3 months	8/61 (13.1)	3 (4.9)	−8.2%	−19.5%	2.6%
6 months	8/59 (13.6)	1 (1.7)	−11.9%	−23.2%	−1.8%

*CI* confidence interval.

At baseline, 12 out of the 70 patients reported at least one day of work loss in the previous 1 month due to asthma (results not shown in table). However, during the study period follow-ups, only 1 patient reported at least one day of work loss at 6-month follow-up.

The proportion that had used the pictorial asthma action plan in the previous month was 58.7% (*n* = 37), 39.3% (*n* = 24) and 33.9% (*n* = 20) at 1, 3 and 6-month follow-ups compared to 21.4% (*n* = 15) who used the written action plan at baseline. The motivations for use included having better asthma control, ease of use and feeling better after using the action plan. Reasons for not using the action plan included 'knowing the action plan by heart', feeling well, and forgetting that they had one.

### Fidelity to delivering the intervention

The fidelity check showed that all participants were recorded as having received counselling on supported self-management and were given the personalised pictorial asthma action plan with relevant medication information by the attending medical officer. All had recorded being given teaching on inhaler technique and medication adherence from the attending pharmacist.

### Cost savings from the use of action plan

The cost-saving analysis was focused on the 59 patients who were available at 6-month follow-up. The estimation of cost saving was based on the differences between the estimated cost incurred in the absence of the intervention and the actual costs as observed. The estimations of the cost incurred in the absence of the intervention (using the action plan) and the actual costs as observed over the 6 months are summarised in Supplementary Information: Cost savings from use of action plan and in Supplementary Tables 2–6). The estimated cost savings from using the action plan for the entire cohort at 6-months follow-up was RM 15,866.22 USD3755.36), and for each patient over the 6 months was 1 RM268.92 (USD63.65), (RM1.00 = USD4.22 on June 6, 2022). Further details on the cost estimated for cost savings from the use of the action plan are described in the Supplementary Information: Cost savings from the use of the action plan.

## Discussion

The pictorial asthma action plan was feasible for use in the Malaysian primary-care setting and has the potential to show clinically significant effects in future studies. With the use of the pictorial asthma action plan, our study has shown an improvement in asthma control at 3- and 6-month follow-ups, and a reduction in acute exacerbations and emergency visits at 3- and 6-months follow-ups, as well as a reduction in hospital admissions at 6-months follow-up. If confirmed in a randomised controlled trial, these improvements have the potential to reduce healthcare costs.

In our study, the doctors, pharmacists and nurses worked together in providing asthma-supported self-management. This is in line with the Malaysian primary healthcare delivery where the goal is to provide comprehensive care by a team comprising doctors, pharmacists, assistant medical officers, and nurses as well as other allied healthcare professionals^37^. To prepare for this, we conducted a 2-h group training session for the doctors and pharmacists on the correct use of the pictorial action plan. We also involved the nurses to make them aware about the use of the action plan in clinical practice. The training included role-plays that incorporated communication skills training with simulated patients, as patient-centred communication has been shown to increase the self-efficacy of the healthcare professionals in delivering counselling^38^.

The recruitment of study participants was facilitated by recruiting participants from the Klang Asthma Cohort registry and enrolment occurred on their follow-up appointment dates for their asthma care at the clinic. The use of a registry enabled timely recruitment and allowed the identification of potential study participants based on eligibility criteria^39^. In addition, enrolment conducted during their scheduled clinic visits facilitated participation with a retention rate of more than 80% at 6-month follow-up. On the other hand, patients who are engaged in the clinical registry may not be typical of people with asthma. They have better commitment towards their healthcare, which will have contributed to the relatively high retention rate observed in our study^40^. In our study, those lost to follow-up were significantly younger and had higher personal income, suggesting that they were more likely to be working or have busy jobs. Uptake of the pictorial asthma action plan and retention in future studies may therefore be improved by offering asthma care during weekends and at private healthcare facilities. From a practical perspective, primary-care clinics could develop their own chronic respiratory disease registry to facilitate identification of patients with limited health literacy to enhance their healthcare.

Asthma control in the present study improved over the 6 months of the study. Similarly, a non-randomised controlled study among women with poor literacy skills who received a pictorial asthma action plan in Turkey showed improvement in asthma control over 6-month duration. In addition, they also found a significant difference at first and second month follow-up when compared to patients who received usual asthma care^21^. However, despite a trend to improvement at 3 months, a trial in Malaysia conducted among patients who attended a semi-urban primary-care clinic did not show a significant difference in asthma control between those who were provided with the action plan and those who received usual care^22^. This could be because their trial had high proportion of patient with good asthma control at baseline.

Our study showed a reduction in the proportion of asthma adults with acute events over the 6 months of the study. This is similar to the findings of ref. ^21^ and reflects the known benefits of optimal self-management that includes education, action plan and regular review and reflects the known benefits of optimal self-management that includes education, action plan and regular review^11,12^. However, as in any before-and-after study, we cannot rule out other factors which may have confounded the intervention effect estimates. Our study was partially conducted during the COVID-19 pandemic, when social distancing and face masks reduced exposure to asthma triggers such as viral infections, environmental pollution and dust. A reduction in acute attacks during the pandemic has been described in the UK^41,42^. In addition, the requirement to ‘work from home’ during the pandemic may have been responsible for the reduced need for medical leave that we observed.

Similar to our findings, optimal asthma control has been associated with reduced total healthcare costs in a longitudinal study conducted among adults with asthma in two primary-care clinics in Singapore^43^. Therefore, any approach incorporated into supported self-management for asthma (such as a pictorial action plan) could not only potentially improve asthma control but also achieve total cost savings for the healthcare system. Our findings were encouraging, but a formal health economic evaluation should be included in a future trial.

This study evaluated the feasibility of a potentially important intervention to improve asthma control among adults with asthma in low- and middle-income countries. We followed a recognised process for content validation of the adapted pictorial asthma action plan, which assessed elements of ‘guessability and translucency’ though not using the methodology employed in the original validation study of the pictorial action plans^20^. Training the healthcare professionals to deliver self-management supported by a pictorial action plan is feasible and may have facilitated better asthma control.

However, there are some limitations. First, a lack of a concurrent control group limits the interpretation to an association and evidence of effectiveness, hence, the need for a randomised controlled trial. Second, we recruited participants via the Klang Asthma Cohort but practices that collaborate with quality improvement research, and their patients who consent to be on a research registry may not be typical of Malaysian primary care and people with asthma. Third, we did not used limited health literacy as an inclusion criterion so we are not able to assess the impact of pictorial plan specifically among those with limited health literacy. However, we are seeking to develop an intervention which can be implemented in routine clinical practice, and formally assessing health literacy prior to providing an action plan is not likely to be normal practice. In the context of high levels of limited health literacy (60%), assuming that a pictorial plan will be most appropriate is likely to be the ‘safest’ option.

The plan does not include the ‘amber’ step of increasing inhaled steroids included in commonly used action plans and does not provide advice for people using combination inhalers. Whilst this may be a limitation, it aligns with the national guideline^10^, which may have enhanced implementation. In addition, whilst emergency steroids are rarely prescribed, advice on when oral steroids are required could prompt timely seeking of medical advice. Future iterations of the action plan will reflect changes in national policy (e.g., use of combination ICS/formoterol as both the reliever and controller as recommended in the current global guidelines^44^). This study will allow informed planning for a future definitive randomised controlled trial.

Fourth, we were not able to verify some secondary outcomes as these were self-reported, hence these results were subjected to recall bias. Next, we found asthma control improved following the used of the action plan, a possible confounder could relate to the COVID-19 pandemic, when social distancing restrictions would have limited exposure to asthma triggers such as circulating viruses, dust or pollens which may have contributed to the improved asthma control observed. In contrast, during the pandemic, the participants’ follow-up and care may be atypical (potentially access was reduced during periods of lockdown), hence, future studies need to consider remote access to healthcare providers. Further, our study was not designed to identify which aspect(s) of asthma self-management are critical to changing behaviour. Finally, because COVID related delays and restrictions, the study duration was limited to 6 months rather than the planned 12 months which may have introduced seasonal biases and also prevented observation of long-term sustainability.

A pictorial asthma action plan for adult asthma patients has the potential to contribute to improving asthma control in the context of Malaysian primary care. This study provides evidence of the feasibility of training primary healthcare professionals and the use of pictorial action plans, suggesting that the intervention has the potential to proceed to a full trial. Furthermore, the pictorial action plan was associated with a favourable impact on asthma control. Future work may include the development of a technology-based intervention for adult asthma patients and a pilot randomised controlled trial, to further improve access.

## Supplementary information

Supplementary Material

Reporting Summary

## Data Availability

The dataset of this study will be held in the Edinburgh DataVault, accessible only to authorised University of Edinburgh staff. Access to the data will be from the Depositor, or in their absence the Contact Person or Data Manager. Further information on retrieving data from the DataVault can be found at http://www.ed.ac.uk/information- services/research-support/research-data-service/sharing-preserving-data/datavault/interim-datavault/retrieve-data. Associated publicly available files will be published at https://datashare.ed.ac.uk/handle/10283/4226/restricted-resource?bitstreamId=727d24b8-7643-4d61-b21e-b8dfccd46bd2.

## Code availability

The codes generated during and/or analysed during this study are available from the corresponding author upon request.
