# Supplementary material for: Feasibility of supported self-management with a pictorial action plan to improve asthma control
Source: NPJ Prim Care Respir Med. 2022 Sep 20;32:34. doi: 10.1038/s41533-022-00294-8 (PMC9486786; doi:10.1038/s41533-022-00294-8)
Supplement: Supplementary file 1 — Supplementary Material [file 41533_2022_294_MOESM1_ESM.pdf]

Supplementary Figure 1

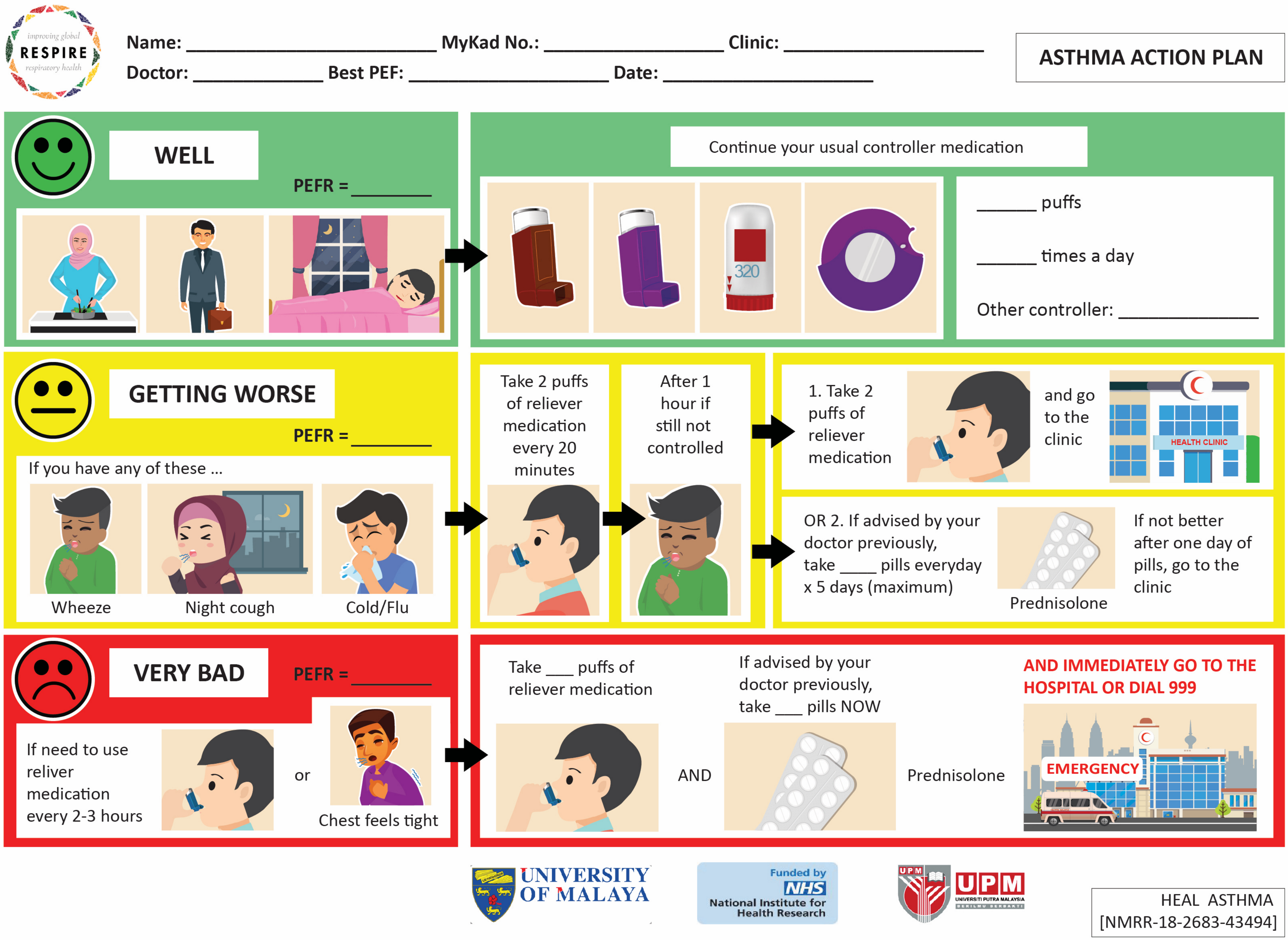

Supplementary Information: Content Validation of the Pictorial Asthma Action Plan

The pictorial action plan underwent content validity checks by nine panelists. The panelists comprised (additional to the stakeholders who helped with the initial development) of four Malaysian adult patients with asthma and five healthcare providers comprising three family physicians, one primary care medical officer (doctor with no postgraduate training, working in primary care clinics under the leadership of specialist family physicians) and one pharmacist. The patients were selected because of the different levels of health literacy. The panelists were asked to comment on the 1) accuracy: the pictures represent the intended meaning of the asthma action plan, 2) clarity: a) the information on the scale for each zone of asthma care was clear; b) pictures were easily understood, 3) style: a) font size was appropriate; b) picture size was appropriate, and 4) relevance of the pictures were relevant to the local social context. The Content Validity Ratio (CVR) was used for validity conformity [1]. Each member of the panel was asked independently to score each item as either essential, not essential or unclear. The formula of $CVR=(N_e - N/2)/(N/2)$, in which N_e is the number of panels indicating "essential" and N is the total number of panels. CVR ranges between 1.00 and -1.00. In this study, there were nine panelists, therefore, if the CVR was at least 0.78, the item regarding the pictorial action plan was considered essential and valid, with a level of significance of 5% [1]. So, for this study, the pictorial action plan was considered essential and valid for use in the Malaysian context as the CVR were 1.00 for all the items except 0.78 for the item on pictures were easily understood. A panelist shared that a picture

representing wheeze was unclear. Hence, based on this feedback to improve clarity we have added in writing the symptoms below.

Reference:

1. Lawshe CH. A quantitative approach to content validity. Personnel Psychology. 1975;564–75.

HEAL ASTHMA – PICTORIAL ASTHMA ACTION PLAN (PRE – POST STUDY) QUESTIONNAIRE - BASELINE DATA

MALAYSIAN ADULT

An NIHR Global Health Research Unit on Respiratory Health (RESPIRE) at the University of Edinburgh project.
www.ed.ac.uk/usher/respire
This research was commissioned by the National Institute of Health Research using Official Development Assistance (ODA) funding. The views expressed are those of the author(s) and not necessarily those of the NHS, the NIHR or the Department of Health and Social Care.

SITE ID:										
DATE OF RECRUITMENT (DD/MM/YYYY):			/			/	2	0		
PATIENT ID:										

SECTION A: PATIENT DEMOGRAPHICS

A1	Age	______ years
A2	Gender	☐ Male<br>☐ Female
A3	Ethnic Group	☐ Malay<br>☐ Chinese<br>☐ Indian<br>☐ Non Malay Bumiputera
A4	Marital status	☐ Single<br>☐ Married<br>☐ Divorced or widower<br>☐ Others (e.g. partners)
A5	Highest educational level attained	☐ No formal education<br>☐ Primary<br>☐ Secondary<br>☐ Tertiary
A6	Occupation	☐ Not working<br>☐ Working<br>☐ Retired<br><br>If working, please state your occupation:<br>______________________<br>☐ Public<br>☐ Private<br>☐ Self-employed
A7	Personal income	RM ____________ /month
A8	Household income	RM ____________ /month
A9	Used smartphone for asthma information	

SECTION B: MEDICAL & PATIENT EXPENDITURE INFORMATION (To be extracted from Klang Asthma Cohort)

B1	Age at diagnosis of asthma by doctor	___________ years
B2	Trigger factors for asthma	☐ Dust<br>☐ Upper respiratory tract infection<br>☐ Smoke<br>☐ Haze<br>☐ Fumes<br>☐ Food<br>☐ Others, please specify _______________________
B3	Family history (up to ***2nd -degree**) of asthma<br>(* **2nd degree family: A person's parents, siblings, children and grandparents)**	☐ No ☐ Yes<br><br>If yes, please state<br>☐ Parents<br>☐ Siblings<br>☐ Children<br>☐ Grandparents
B4	History of allergy	☐ No ☐ Yes<br><br>If yes, please state<br>☐ Food<br>☐ Drugs<br>☐ Animal fur<br>☐ Others, please specify: _____________________
B5	Current smoking status<br><br>If yes, forms of smoking<br><br><br><br><br><br>Age starts smoking<br><br>On average, how many cigarettes do you smoke per day?<br>If no, are you an ex-smoker?<br><br>If yes, age starts smoking<br><br>Duration of smoking	☐ No ☐ Yes<br><br>☐ Cigarette<br>☐ Pipes<br>☐ Cigar<br>☐ e-cigarette<br>☐ Others, please specify: _____________________<br><br>________ years<br><br>_________ sticks per day<br><br>☐ No ☐ Yes<br><br>_________ years<br><br>_________ years

B6	Do you have any medical conditions?	☐ No ☐ Yes<br><br>If yes, please state<br>☐ Diabetes<br>☐ Hypertension<br>☐ Dyslipidemia<br>☐ Cardiac disorders<br>☐ Allergic rhinitis<br>☐ Obesity<br>☐ Others, please specify: ____________________
B7	Have you ever used any other medications/treatment for asthma besides western medications (e.g.: complementary/traditional/ alternative medicines)?<br>if yes, please specify:	☐ No ☐ Yes<br><br>☐ Animal products<br>☐ Chinese herbs<br>☐ Malay herbs<br>☐ Indian herbs<br>☐ Physical treatment<br>☐ Not sure<br>☐ Others, please specify: ____________________
B8	Have you ever received the following vaccinations?<br>a. Pneumococcal in the last 5 years<br><br>b. Influenza in the last 1 year	 <br>☐ No ☐ Yes<br><br>☐ No ☐ Yes
B9	Asthma medications<br>a) In the past 1 month, have you ever used reliever medication?<br><br>If yes, please specify about the reliever<br><br>How many times?<br><br>b) In the past 1 month, have you ever missed your controller medications?<br>If yes, how often have you missed controller medications?	☐ No ☐ Yes<br><br>☐ Salbutamol tablets<br>☐ Salbutamol inhaler<br><br>_______ times<br><br>☐ No ☐ Yes<br><br>_______ times
B10	Do you have written instruction from your doctor on how to manage your asthma if it gets worse or if you have an attack (asthma action plan)?	☐ No ☐ Yes ☐ Not sure

	If yes; do you use it?	☐ No ☐ Yes
B11	Do you have an asthma diary?<br><br>If yes; do you record most times?	☐ No ☐ Yes ☐ Not sure<br><br>☐ No ☐ Yes
B12	Have you ever received asthma education from any clinic staff? *clinic staff include all medical personnel	☐ No ☐ Yes ☐ Not sure
B13	In the last 1 month, have you had any ***exacerbation/attack**?<br><br>If yes, how many times in the last 1 month?	☐ No ☐ Yes<br><br>_________ times
	***Exacerbation: Episodes characterised by acute or subacute onset of progressively worsening symptoms, such as shortness of breath, cough, wheezing or chest tightness, which are worse than the patient's usual status and require a change in treatment.**	
B14	In the past 1 month, have you had any emergency visit for asthma?<br><br>If yes, how many times of emergency visit for asthma?	☐ No ☐ Yes<br><br>_________ times
B15	In the past 1 month, have you been admitted to any hospital admission for asthma?<br><br>If yes, how many times admitted?	☐ No ☐ Yes<br><br>_________ times
B16	On average in 1 month, how many days do you need to take your own leave(s) for asthma treatment?	_________ days per month
B17	On average in 1 month, how many days of medical certificate did you receive for asthma?	_________ days per month

SECTION C: GINA ASTHMA SYMPTOM CONTROL

In the past 4 week, do you have:			Well controlled	Partly controlled	Uncontrolled
• Daytime asthma symptoms more than twice/week	☐ No	☐ Yes	None of these	1 – 2 of these	3 – 4 of these
• Any night waking due to asthma?	☐ No	☐ Yes			
• Reliever needed for symptoms more than twice/week	☐ No	☐ Yes			
• Any activity limitation due to asthma?	☐ No	☐ Yes			

SECTION D: HEALTH LITERACY SURVEY-ASIA-Q47

On a scale from very easy to very difficult, how easy would you say it is to:

		Very difficult	Difficult	Easy	Very easy
Q 1	…find information about symptoms of illnesses that concern you?				
Q 2	…find information on treatments of illnesses that concern you?				
Q 3	…find out what to do in case of a medical emergency?				
Q 4	…find out where to get professional help (such as doctor, pharmacist, psychologist) when you are ill?				
Q 5	…understand what your doctor says to you?				
Q 6	…understand the leaflets that come with your medicine?				
Q 7	…understand what to do in a medical emergency?				
Q 8	…understand your doctor's or pharmacist's instruction on how to take a prescribed medicine?				
Q 9	…judge how information from your doctor applies to you?				
Q 10	…judge the advantages and disadvantages of different treatment options?				
Q 11	…judge when you may need to get a second opinion from another doctor?				
Q 12	…judge if the information about illness in the media (such as TV, Internet, or other media) is reliable?				
Q 13	…use information the doctor gives you to make decisions about your illness?				
Q 14	…follow the instructions on medication?				
Q 15	…call an ambulance in an emergency?				
Q 16	…follow instructions from your doctor or pharmacist?				
Q 17	…find information about how to manage unhealthy behavior such as smoking, low physical activity and drinking too much?				
Q 18	…find information on how to manage mental health problems like stress or depression?				
Q 19	…find information about vaccinations and health screenings (such as breast exam, blood sugar test, blood pressure) that you should have?				
Q 20	…find information on how to prevent or manage conditions like being overweight, high blood pressure or high cholesterol?				
Q 21	…understand health warnings about behavior such as smoking, low physical activity and drinking too much?				
Q 22	…understand why you need vaccinations?				
Q 23	…understand why you need health screenings (such as breast exam, blood sugar test, blood pressure)?				
Q 24	…judge how reliable health warnings are, such as smoking, low physical activity and drinking too much?				

Q 25	…judge when you need to go to a doctor for a check-up?				
Q 26	…judge which vaccinations you may need?				
Q 27	…judge which health screenings (such as breast exam, blood sugar test, blood pressure) you should have?				
Q 28	…judge if the information on health risks in the media (such as TV, Internet or other media) is reliable?				
Q 29	…decide if you should have a flu vaccination?				
Q 30	…decide how you can protect yourself from illness based on advice from family and friends?				
Q 31	…decide how you can protect yourself from illness based on information in the media (such as Newspaper, leaflets, Internet or other media)?				
Q 32	…find information on healthy activities such as exercise, healthy food and nutrition?				
Q 33	…find out about activities (such as meditation, exercise, walking, Pilates etc.) that are good for your mental well-being?				
Q 34	…find information (such as reducing noise and pollution, creating green spaces, leisure facilities) on how your neighborhood could be more health-friendly?				
Q 35	…find out about political changes (such as legislation, new health screening programs, change of government, restructuring of health services etc.) that may affect health?				
Q 36	…find out about efforts to promote your health at work?				
Q 37	…understand advice on health from family members or friends?				
Q 38	…understand information on food packaging?				
Q 39	…understand information in the media (such as Internet, newspaper, magazines) on how to get healthier?				
Q 40	…understand information on how to keep your mind healthy?				
Q 41	…judge how where you live (such as your community, neighborhood) affects your health and well-being?				
Q 42	…judge how your housing conditions help you to stay healthy?				
Q 43	…judge which everyday behavior (such as drinking and eating habits, exercise etc.) is related to your health?				
Q 44	…make decisions to improve your health?				
Q 45	… join a sports club or exercise class if you want to?				
Q 46	…influence your living conditions that affect your health and well being?				
Q 47	…take part in activities that improve health and well-being in your community?				

HEAL ASTHMA – PICTORIAL ASTHMA ACTION PLAN (PRE – POST STUDY) QUESTIONNAIRE – FOLLOW UP

MALAYSIAN ADULT

An NIHR Global Health Research Unit on Respiratory Health (RESPIRE) at the University of Edinburgh project.
www.ed.ac.uk/usher/respire
This research was commissioned by the National Institute of Health Research using Official Development Assistance (ODA) funding. The views expressed are those of the author(s) and not necessarily those of the NHS, the NIHR or the Department of Health and Social Care.

SITE ID:										
DATE OF CALL (DD/MM/YYYY):			/			/	2	0		
PATIENT ID:										

Case report form: At 1/3/6 month follow-up

SECTION A: ASTHMA ACTION PLAN (please circle the number indicating answer or fill in the blanks)

Question		Answer
A1	Have been using the asthma action plan?<br>**If no, proceed to A2**<br>If yes, how frequent do you use the action plan in a week?<br><br>May we know your motivations to use the action plan?	☐ No ☐ Yes<br><br>________ times<br><br>☐ To have good asthma control<br>☐ I feel better<br>☐ I can breath better<br>☐ I now understand the purpose of the asthma action plan<br>☐ It is easy to use<br>☐ Family support<br>☐ Other reasons, please specify<br>________________________
A2	May we know the reason for not using the asthma action plan?	☐ Not useful<br>☐ Lost it<br>☐ Too difficult to use<br>☐ I feel well<br>☐ Other reasons, please specify<br>________________________
A3	Were your asthma medications changed over this past one month?<br>If yes, what were changed?	☐ No ☐ Yes<br><br>________________________
A4	Asthma medications<br>a) In the past 1 month, have you ever used reliever medication?<br>If yes, please specify about the reliever	☐ No ☐ Yes<br><br>☐ Salbutamol tablets<br>☐ Salbutamol inhaler

	How many times in the last 1 month?<br><br>b) In the past 1 month, have you ever missed your controller medications?<br>If yes, how often have you missed controller medications in the last 1 month?	_______ times<br><br>☐ No ☐ Yes<br><br>_______ times
A5	In the last 1 month, have you had any ***exacerbation/attack**?<br><br>If yes, how many times?	☐ No ☐ Yes<br><br>_________ times
A6	In the past 1 month, have you had any emergency visit for asthma?<br><br>If yes, how many times of emergency visit for asthma?	☐ No ☐ Yes<br><br>_________ times
A7	In the past 1 month, have you been admitted to any hospital admission for asthma?<br><br>If yes, how many times admitted?	☐ No ☐ Yes<br><br>_________ times
A8	On average in 1 month, how many days do you need to take your own leave(s) for asthma treatment?	_________ days per month
A9	On average in 1 month, how many days of medical certificate did you receive for asthma?	_________ days per month

SECTION B: ASTHMA CONTROL QUESTIONNAIRE

In the past 4 week, do you have:			Well controlled	Partly controlled	Uncontrolled
• Daytime asthma symptoms more than twice/week	☐ No	☐ Yes			
• Any hight waking due to asthma?	☐ No	☐ Yes	None of these	1 – 2 of these	3 – 4 of these
• Reliever needed for symptoms more than twice/week	☐ No	☐ Yes			
• Any activity limitation due to asthma?	☐ No	☐ Yes			

An NIHR Global Health Research Unit on Respiratory Health (RESPIRE) at the University of Edinburgh project.
www.ed.ac.uk/usher/respire
This research was commissioned by the National Institute of Health Research using Official Development Assistance (ODA) funding. The views expressed are those of the author(s) and not necessarily those of the NHS, the NIHR or the Department of Health and Social Care.

UNIVERSITY OF MALAYA

Funded by NHS National Institute for Health Research

UPM UNIVERSITI PUTRA MALAYSIA BERILMU BERBAKTI

Supplementary Information: Cost of asthma related care

The detailed costs of asthma related care are listed in Supplementary Table 1. The underlying costs were based on the Government of Malaysia Fee Schedule[35] and were adjusted to the 2019 costs. In addition, the distribution of the proportions of the cost shared among patients for outpatient visits, investigations and medications were based on expert opinion (comprising Ministry of Health (MoH) family medicine specialist and a pharmacist, and the research team who were family medicine specialists and a respiratory physician).

Supplementary Table 1: Details of costs for outpatient visits, investigations, treatments and admission

	Category of Service	Item	Share among patients	Monthly usage	Unit Cost (2014 RM)	Unit Cost (2019 RM)	Total Cost (2019 RM)
1	**Outpatient visit to a public clinic**						**68.90 (USD15.66)**
		General outpatient	70%		40.00	43.06	
		Specialist outpatient	30%		120.00	129.18	
2	**Outpatient visit to a public hospital**						**109.80 (USD24.96)**
		Specialist outpatient	10%		120.00	129.18	
		Emergency department	90%		100.00	107.65	
3	**Investigations related to outpatient visit**						**139.95 (USD31.81)**
		Chest X-ray			60.00	64.59	
		Spirometry			70.00	75.36	
4	**Medication prescribed to outpatients on monthly basis**						
	**Well-controlled patient**						**10.08 (USD2.29)**
		Salbutamol inhaler		25%		5.97	
		Budesonide inhaler		40%		21.46	
	**Partly-controlled patient**						**27.43 (USD6.24)**
		Salbutamol inhaler		100%		5.97	
		Budesonide inhaler		100%		21.46	

	Category of Service	Item	Share among patients	Monthly usage	Unit Cost (2014 RM)	Unit Cost (2019 RM)	Total Cost (2019 RM)
	**Poorly-controlled patient**						**27.43 (USD6.24)**
		Salbutamol inhaler		100%		5.97	
		Budesonide inhaler		100%		21.46	
5	**Admission per day**						**301.42 (USD68.52)**
		Ward fee			180.00	193.77	
		Treatment fee			100.00	107.65	
6	**Investigations per admission**						**229.30 (USD52.12)**
		Chest X-ray			60.00	64.59	
		Renal profile			38.00	40.91	
		Arterial blood gases			65.00	69.97	
		Full blood count			40.00	43.06	
		Random blood glucose			10.00	10.77	

RM1.00=USD4.40 on 20 June 2022

Supplementary Information: Cost savings for asthma related care

The cost savings analysis was made based on the 59 participants that complete the study at 6-month follow-up. The estimation on cost savings for asthma related care was made on the following assumptions as shown in Supplementary Table 2.

Supplementary Table 2: Assumptions on cost savings for asthma related care

No.	Assumptions
1.	The cost incurred in the absence of the intervention was estimated based on the asthma status of participants at baseline remains throughout the 6 months of follow-up.
2.	Costs for well-controlled participants was estimated from participants who were assessed as well-controlled at both baseline and at 6 months follow-up.
3.	Costs for uncontrolled participants estimated from participants who were assessed as uncontrolled at both baseline and at 6 months follow-up.
4.	Estimation of costs combined:<br>a. resource use as obtained from the study<br>b. unit costs obtained from published government fee schedule and consensus from expert panel
5.	It was assumed that all hospital admissions and clinic visits were in the public Ministry of Health (MoH) facilities. The components of costs included in this estimation were:<br>a. investigations, treatment and ward charges for hospital admissions<br>b. charges for emergency hospital visits<br>c. charges for outpatient visits to MoH clinics and specialist outpatient clinics (SOPCs) in MoH hospitals<br>d. investigations done for outpatients in SOPCs in MoH hospitals<br>e. medications (costed separately for well-controlled and uncontrolled participants).
6.	Resource use had only been captured for 3 months out of the 6 months follow up (for months 1, 3 and 6 during the follow up at 1 month, 3 month and 6 month). It is assumed that the average resource use per participant per month during these 3 months represent the monthly resource use throughout the 6 months of study.
7.	In order, to estimate medication costs, it is assumed that:<br>a. asthma status at baseline remains for month 1<br>b. asthma status at 1 month follow-up remains for months 2 and 3<br>c. asthma status at 3 month follow-up remains for months 4, 5 and 6

The costs for hospital admissions, emergency visits and outpatient clinic visits and medications were calculated for both well-controlled and uncontrolled participants at each follow-up time points as summarised in Supplementary Table 3. These costs were estimated based on the detailed costing as presented in the Online Supplementary Table 1. Supplementary Table 4 is the summary of costs for medications at baseline and at 1- and 3-month follow ups.

Supplementary Table 3: Summary of costs for hospital admissions, emergency visits and outpatient clinic visits

	Overall			Well-controlled throughout			Uncontrolled throughout		
	1-month follow-up	3-month follow-up	6-month follow-up	1-month follow-up	3-month follow-up	6-month follow-up	1-month follow-up	3-month follow-up	6-month follow-up
	N=59	N=59	N=59	N=16	N=16	N=16	N=19	N=19	N=19
**Hospital admissions**									
No. of patients admitted	0	3	1	0	1	0	0	2	1
Total admissions	0	3	1	0	1	0	0	2	1
Costs of inpatient investigation (monthly)	RM0.00	RM687.89	RM229.30	RM0.00	RM229.30	RM0.00	RM0.00	RM458.59	RM229.30
Total number of days admitted	0	13	1	0	3	0	0	10	1
Costs for ward and treatment (monthly)	RM0.00	RM3,918.48	RM301.42	RM0.00	RM904.27	RM0.00	RM0.00	RM3,014.22	RM301.42
**Total hospitalisation costs/ participant (monthly)**	**RM0.00**	**RM78.07**	**RM9.00**	**RM0.00**	**RM70.85**	**RM0.00**	**RM0.00**	**RM182.78**	**RM27.93**
**Emergency visits**									
No. of patients	4	8	8	0	1	2	4	4	4
Total number of visits	4	9	11	0	1	3	4	5	6
Costs for emergency visit (monthly)	RM430.60	RM968.86	RM1,184.16	RM0.00	RM107.65	RM322.95	RM430.60	RM538.25	RM645.90

	Overall			Well-controlled throughout			Uncontrolled throughout		
**Costs for emergency visit/participant (monthly)**	**RM7.30**	**RM16.42**	**RM20.07**	**RM0.00**	**RM6.73**	**RM20.18**	**RM22.66**	**RM28.33**	**RM33.99**
	**1-month follow-up**	**3-month follow-up**	**6-month follow-up**	**1-month follow-up**	**3-month follow-up**	**6-month follow-up**	**1-month follow-up**	**3-month follow-up**	**6-month follow-up**
**Outpatient visits**									
No. of patients	10	8	22	1	0	8	6	5	8
Total number of visits	10	11	24	1	0	8	6	6	10
MoH clinic	8	9	24	1	0	8	6	6	10
MoH hospitals	2	2	0	0	0	0	0	2	0
Costs for outpatient visit to MoH clinic (monthly)	RM551.17	RM620.07	RM1,653.51	RM68.90	RM0.00	RM551.17	RM413.38	RM413.38	RM688.96
Costs for outpatient visit to MoH hospital (monthly)	RM258.36	RM258.36	RM0.00	RM0.00	RM0.00	RM0.00	RM0.00	RM258.36	RM0.00
Costs of outpatient investigation in specialist outpatient clinic (monthly)	RM0.00	RM0.00	RM0.00	RM0.00	RM0.00	RM0.00	RM0.00	RM0.00	RM0.00
**Costs for outpatient visits/ participant (monthly)**	**RM13.72**	**RM14.89**	**RM28.03**	**RM4.31**	**RM0.00**	**RM34.45**	**RM21.76**	**RM35.35**	**RM36.26**

Note: MoH=Ministry of Health

Supplementary Table 4: Summary of costs for medications at baseline, 1- and 3-month follow ups

	Overall			Well-controlled throughout			Not controlled throughout		
	Baseline	1-month follow-up	3-month follow-up	Baseline	1-month follow-up	3-month follow-up	Baseline	1-month follow-up	3-month follow-up
No. of well-controlled participants	18	26	30	16	16	16	0	0	0
No. of uncontrolled participants	41	33	29	0	0	0	19	19	19
Cost for medication (monthly)	RM1,124.63	RM1,167.18	RM1,097.77	RM161.22	RM161.22	RM161.22	RM521.17	RM521.17	RM521.17
**Cost for medication/ participant (monthly)**	**RM19.06 (USD4.33)**	**RM19.78 (USD4.50)**	**RM18.61 (USD4.23)**	**RM10.08 (USD2.29)**	**RM10.08 (USD2.29)**	**RM10.08 (USD2.29)**	**RM27.43 (USD6.24)**	**RM27.43 (USD6.24)**	**RM27.43 (USD6.24)**

RM1.00=USD4.40 on 20 June 2022

The estimation of cost saving was based on the differences between the estimated cost incurred in the absence of the intervention and the actual costs as observed. The estimations of cost incurred in the absence of the intervention (using the action plan) and the actual costs as observed over the 6 months are summarised in Supplementary Table 5 and 6, respectively. The estimated savings from using the action plan for the entire cohort and for each patient over the 6 months was RM 15,866.22 (USD3,606.80) and RM268.92 (USD61.13) (RM1.00=USD4.40 on 20 June 2022), respectively.

Supplementary Table 5: Estimations of cost incurred in the absence of the intervention

No.	Description	Well-controlled	Uncontrolled	Total
1	No. of patients	18	41	59
2	Duration in months	6	6	6
3	Costs for hospital admissions	RM2,550.51	RM17,278.38	RM19,828.89
4	Costs for emergency visits	RM968.86	RM6,968.96	RM7,937.82
5	Costs for outpatient clinic visits	RM1,395.15	RM8,864.52	RM10,259.67
6	Costs for medications	RM1,088.26	RM6,747.78	RM7,836.04
	**Total Cost**			**RM45,862.43 (USD10,425.72)**

RM1.00=USD4.40 on 20 June 2022

Supplementary Table 6: Estimations of the actual costs as observed

No.	Description	Total
1	No. of patients	59
2	Duration in months	6
3	Costs for hospital admissions	RM10,274.18
4	Costs for emergency visits	RM5,167.23
5	Costs for outpatient clinic visits	RM7,802.52
6	Costs for medications	RM6,752.28
	**Total Cost**	**RM29,996.21 (USD6,818.92)**

RM1.00=USD4.40 on 20 June 2022
