# Supplementary material for: Feasibility of supported self-management with a pictorial action plan to improve asthma control
Source: NPJ Prim Care Respir Med. 2022 Sep 20;32:34. doi: 10.1038/s41533-022-00294-8 (PMC9486786; doi:10.1038/s41533-022-00294-8)
Supplement: Supplementary file 2 — Reporting Summary [file 41533_2022_294_MOESM2_ESM.pdf]

nature portfolio

Corresponding author(s): Shariff Ghazali, Sazlina

Last updated by author(s): Jul 20, 2022

Reporting Summary

Nature Portfolio wishes to improve the reproducibility of the work that we publish. This form provides structure for consistency and transparency in reporting. For further information on Nature Portfolio policies, see our Editorial Policies and the Editorial Policy Checklist.

Statistics

For all statistical analyses, confirm that the following items are present in the figure legend, table legend, main text, or Methods section.

n/a	Confirmed	
☐	☒	The exact sample size (*n*) for each experimental group/condition, given as a discrete number and unit of measurement
☐	☒	A statement on whether measurements were taken from distinct samples or whether the same sample was measured repeatedly
☐	☒	The statistical test(s) used AND whether they are one- or two-sided<br>*Only common tests should be described solely by name; describe more complex techniques in the Methods section.*
☐	☒	A description of all covariates tested
☐	☒	A description of any assumptions or corrections, such as tests of normality and adjustment for multiple comparisons
☐	☒	A full description of the statistical parameters including central tendency (e.g. means) or other basic estimates (e.g. regression coefficient) AND variation (e.g. standard deviation) or associated estimates of uncertainty (e.g. confidence intervals)
☐	☒	For null hypothesis testing, the test statistic (e.g. *F*, *t*, *r*) with confidence intervals, effect sizes, degrees of freedom and *P* value noted<br>*Give P values as exact values whenever suitable.*
☒	☐	For Bayesian analysis, information on the choice of priors and Markov chain Monte Carlo settings
☒	☐	For hierarchical and complex designs, identification of the appropriate level for tests and full reporting of outcomes
☒	☐	Estimates of effect sizes (e.g. Cohen's *d*, Pearson's *r*), indicating how they were calculated

Our web collection on statistics for biologists contains articles on many of the points above.

Software and code

Policy information about availability of computer code

Data collection	No software was used for data collection
Data analysis	We used IBM SPSS version 26.0 and R software version 4.0.4, for the statistical analysis. The codes generated during and/or analysed during the current study are available from the corresponding author upon reasonable request.

For manuscripts utilizing custom algorithms or software that are central to the research but not yet described in published literature, software must be made available to editors and reviewers. We strongly encourage code deposition in a community repository (e.g. GitHub). See the Nature Portfolio guidelines for submitting code & software for further information.

Data

Policy information about availability of data

All manuscripts must include a data availability statement. This statement should provide the following information, where applicable:

- Accession codes, unique identifiers, or web links for publicly available datasets
- A description of any restrictions on data availability
- For clinical datasets or third party data, please ensure that the statement adheres to our policy

The dataset of this study will be held in the Edinburgh DataVault, accessible only to authorised University of Edinburgh staff. Access to the data will be from the Depositor, or in their absence the Contact Person or Data Manager. Further information on retrieving data from the DataVault can be found at: http://www.ed.ac.uk/information- services/research-support/research-data-service/sharing-preserving-data/datavault/interim-datavault/retrieve-data. Associated

publicly available files will be published at https://datashare.ed.ac.uk/handle/10283/4226/restricted-resource?bitstreamId=727d24b8-7643-4d61-b21e-b8dfccd46bd2

Human research participants

Policy information about studies involving human research participants and Sex and Gender in Research.

Reporting on sex and gender	We collected data on gender which was self-reported by the participants and we only analysed the gender data to describe the study's participants characteristics
Population characteristics	The mean age of the participants was 51.2 SD15.5 years, 58.6% were women, 72.3% were married and 48.6% were Indian, 42.9% were Malay and 8.6% with Chinese and other ethnicities. The majority (91.4%) of the participants had previously received supported self-management asthma education from the clinic staff, 25 (35.7%) had a written action plan of whom only 60% had used the plan. Even though 54 (77.1%) had an asthma diary, only 14 of them used it. About one-fifth of the participants used complementary and alternative medicines. In terms of health literacy, 61.4% had limited literacy. Only 24.3% used a smartphone for asthma information.
Recruitment	The study participants were recruited from one of the primary care clinics under the Klang Asthma Cohort (KAC) registry (a clinical asthma patients registry) using an Excel generated simple random table by a research member, based on the inclusion and exclusion criteria in Table 1. Klang Asthma Cohort is a cohort of 1,280 people with asthma recruited from six primary care clinics in Klang who are willing to be approached for future research. Detailed description on KAC can be accessed at https://www.ed.ac.uk/usher/respire/chronic-respiratory-disorders/asthma-care.
Ethics oversight	Regulatory approvals have been obtained in line with the operating procedures of the RESPIRE Global Unit including approvals from the National Medical Research Ethics Committee, Ministry of Health, Malaysia [NMRR-18-2683-43494] and relevant authorities involved in the Klang District. Both verbal and written informed consent were obtained from eligible participants before involvement of this study. Confidentiality of the participants was ensured; data was anonymised before publication or report writing. The study was conducted in accordance with the principles of the International Conference on Harmonisation Tripartite Guideline for Good Clinical Practice. This study also received sponsorship approval from the Academic and Clinical Central Office for Research & Development (ACCORD) at the University of Edinburgh.

Note that full information on the approval of the study protocol must also be provided in the manuscript.

Field-specific reporting

Please select the one below that is the best fit for your research. If you are not sure, read the appropriate sections before making your selection.

☒ Life sciences ☐ Behavioural & social sciences ☐ Ecological, evolutionary & environmental sciences

For a reference copy of the document with all sections, see nature.com/documents/nr-reporting-summary-flat.pdf

Life sciences study design

All studies must disclose on these points even when the disclosure is negative.

Sample size	As this was a feasibility study, a formal sample size calculation was not required. Seventy participants were recruited, which was deemed to be adequate to inform the feasibility of delivering the intervention (Teare et. al., 2014)
Data exclusions	There was no data excluded from the analyses.
Replication	Not applicable as the current study is not experimental.
Randomization	The study participants were recruited from one of the primary care clinic under the Klang Asthma Cohort (KAC) registry (a clinical asthma patients registry) using an Excel generated simple random table by a research member, based on the inclusion and exclusion criteria.
Blinding	Not applicable

Reporting for specific materials, systems and methods

We require information from authors about some types of materials, experimental systems and methods used in many studies. Here, indicate whether each material, system or method listed is relevant to your study. If you are not sure if a list item applies to your research, read the appropriate section before selecting a response.

Materials & experimental systems

n/a	Involved in the study
☒	☐ Antibodies
☒	☐ Eukaryotic cell lines
☒	☐ Palaeontology and archaeology
☒	☐ Animals and other organisms
☐	☒ Clinical data
☒	☐ Dual use research of concern

Methods

n/a	Involved in the study
☒	☐ ChIP-seq
☒	☐ Flow cytometry
☒	☐ MRI-based neuroimaging

Clinical data

Policy information about clinical studies

All manuscripts should comply with the ICMJE guidelines for publication of clinical research and a completed CONSORT checklist must be included with all submissions.

Clinical trial registration

ISRCTN87128530; Prospectively registered: 5 September 2019, http://www.isrctn.com/ISRCTN87128530.

Study protocol

The study protocol can be accessed at http://www.isrctn.com/ISRCTN87128530.

Data collection

Embedded within the Medical Research Council framework for design and evaluation of complex interventions23, this pre-post feasibility study was conducted in an urban public primary care clinic in the district of Klang, Selangor, Malaysia between September 2019 to July 2020. The study protocol was registered with BMC ISRCTN Registry [ISRCTN87128530; Prospectively registered: 5 September 2019, http://www.isrctn.com/ISRCTN87128530]. The state of Selangor was chosen as it has a high prevalence of adults with asthma (22%)3, especially in urban communities as well as the highest prevalence of limited health literacy in Malaysia at 75%. Data were collected face-to-face using a pretested structured questionnaire in English or Malay language at baseline. Follow-up data on all the primary and secondary outcomes were collected at 1-, 3- and 6-month post-intervention by trained enumerators who were medical doctors not involved in patients' recruitment and baseline assessments. At every follow up visit, primary and secondary outcomes were collected and participants asked about reasons for using a pictorial action plan and any barriers and facilitators.

Outcomes

Primary outcome
Asthma control was measured using the validated Global Initiative for Asthma (GINA) Asthma Symptoms Control9. This questionnaire comprises four questions that measure the adequacy of asthma treatment in the past four weeks. The questions focus on day and night-time symptoms, use of reliever, and limitation of activity due to asthma. The option for each response is either "Yes" or "No". Well-controlled was considered if the responses to all questions were "No". Any responses of "Yes" were considered as not controlled.

Secondary outcomes
The secondary outcomes measured in this study all related to the previous one-month:
• Number of times reliever medication (inhaled or oral bronchodilators) was used
• Adherence to controller medication
• Frequency of acute exacerbations (defined as episodes characterised by acute or subacute onset of progressively worsening symptoms, such as shortness of breath, cough, wheezing or chest tightness, which are worse than the patient's usual status and require a change in treatment)
• Frequency of asthma-related emergency visits (to a health clinic and/or hospital emergency department)
• Frequency of asthma-related admissions
• Numbers of days lost from work for asthma treatment (defined as number of days of medical leave taken by an employee, or unable to work if self-employed)
• Number of times the participants reported using their pictorial asthma action plan in the previous month
Primary outcome
Asthma control was measured using the validated Global Initiative for Asthma (GINA) Asthma Symptoms Control9. This questionnaire comprises four questions that measure the adequacy of asthma treatment in the past four weeks. The questions focus on day and night-time symptoms, use of reliever, and limitation of activity due to asthma. The option for each response is either "Yes" or "No". Well-controlled was considered if the responses to all questions were "No". Any responses of "Yes" were considered as not controlled.

Secondary outcomes
The secondary outcomes measured in this study all related to the previous one-month:
• Number of times reliever medication (inhaled or oral bronchodilators) was used
• Adherence to controller medication
• Frequency of acute exacerbations (defined as episodes characterised by acute or subacute onset of progressively worsening symptoms, such as shortness of breath, cough, wheezing or chest tightness, which are worse than the patient's usual status and require a change in treatment)
• Frequency of asthma-related emergency visits (to a health clinic and/or hospital emergency department)
• Frequency of asthma-related admissions
• Numbers of days lost from work for asthma treatment (defined as number of days of medical leave taken by an employee, or unable to work if self-employed)
• Number of times the participants reported using their pictorial asthma action plan in the previous month
